# Transposable element distribution, abundance and role in genome size variation in the genus *Oryza*

**DOI:** 10.1186/1471-2148-7-152

**Published:** 2007-08-29

**Authors:** Andrea Zuccolo, Aswathy Sebastian, Jayson Talag, Yeisoo Yu, HyeRan Kim, Kristi Collura, Dave Kudrna, Rod A Wing

**Affiliations:** 1 Arizona Genomics Institute, Department of Plant Sciences, BIO5 Institute, University of Arizona, Tucson, AZ 85721, USA

## Abstract

**Background:**

The genus *Oryza *is composed of 10 distinct genome types, 6 diploid and 4 polyploid, and includes the world's most important food crop – rice (*Oryza sativa *[AA]). Genome size variation in the *Oryza *is more than 3-fold and ranges from 357 Mbp in *Oryza glaberrima *[AA] to 1283 Mbp in the polyploid *Oryza ridleyi *[HHJJ]. Because repetitive elements are known to play a significant role in genome size variation, we constructed random sheared small insert genomic libraries from 12 representative *Oryza *species and conducted a comprehensive study of the repetitive element composition, distribution and phylogeny in this genus. Particular attention was paid to the role played by the most important classes of transposable elements (Long Terminal Repeats Retrotransposons, Long interspersed Nuclear Elements, helitrons, DNA transposable elements) in shaping these genomes and in their contributing to genome size variation.

**Results:**

We identified the elements primarily responsible for the most strikingly genome size variation in *Oryza*. We demonstrated how Long Terminal Repeat retrotransposons belonging to the same families have proliferated to very different extents in various species. We also showed that the pool of Long Terminal Repeat Retrotransposons is substantially conserved and ubiquitous throughout the *Oryza *and so its origin is ancient and its existence predates the speciation events that originated the genus. Finally we described the peculiar behavior of repeats in the species *Oryza coarctata *[HHKK] whose placement in the *Oryza *genus is controversial.

**Conclusion:**

Long Terminal Repeat retrotransposons are the major component of the *Oryza *genomes analyzed and, along with polyploidization, are the most important contributors to the genome size variation across the *Oryza *genus. Two families of Ty3-*gypsy *elements (*RIRE2 *and *Atlantys*) account for a significant portion of the genome size variations present in the *Oryza *genus.

## Background

The C-value paradox, the phenomenon describing the lack of correlation between biological complexity of an organism and its genome size [1], is probably best exemplified in plants were genome sizes span several orders of magnitude ranging from the 98 Mbp *Fragaria viridis *genome [2] to more than 110,000 Mbp genome of the lily *Fritillaria assiriaca *[3]. Significant genome size variations are also not uncommon even within single genera such as rice, cotton and sorghum were 3.6, 3 and 8.1 fold genome size variation have been reported, respectively [4-6]. The primary mechanisms that contribute to this variation in plants are polyploidization [7,8] and transposable element (TE) proliferation [9-11] and elimination [12-17]. TEs are classified according to the different molecules used as intermediates in the replicative mechanism of transposition as class 1(or RNA elements) and class 2 (or DNA elements) [18]. Class 1 elements transpose *via *an RNA intermediate and include long terminal repeat (LTR) retrotransposons (LTR-RTs), long interspersed nuclear elements (LINEs) and short interspersed nuclear elements (SINEs) [19]. Class 2 elements transpose *via *a DNA intermediate and have been classified into superfamilies (hAT, CACTA and *Mutator*-like elements) according to the similarity of transposases, the element-encoded protein that catalyzes transposition and integration [20]. Other classes of DNA transposable elements are represented by helitrons [21] and polintons [22].

The genus *Oryza *is an ideal model system to study the role of TEs on genome size variation. The genus is composed of 23 species: two cultivated (*Oryza sativa *and *Oryza glaberrima*) and 21 wild [23,24]. Based on evidence derived from interspecific crossing, cytogenetics and genomic DNA hybridization, ten *Oryza *genome types have been recognized including 6 diploid (2n = 24) and 4 tetraploid (2n = 48). The relevance of rice as staple food resource for the world, its compact 389 Mbp genome [25] and its role as a "model" species for genomic studies of cereals [26,27] have driven massive research efforts that include the production of the first finished genome sequence of any crop plant [25]. Additionally, our laboratory has developed a comprehensive set of BAC libraries, BAC end sequences and integrated physical maps representing the 10 *Oryza *genome types [28,29].

To better understand the role transposable elements have played in genome size variation in *Oryza *we generated and sequenced a set of random sheared genomic libraries from 12 species representative of the 10 genome types of the *Oryza*. Random sheared libraries represent an unbiased sampling of genome content and enable the characterization of their most relevant features without undertaking a massive sequencing effort. The sequences obtained were analyzed in order to describe, classify and compare the repetitive fraction of the genus *Oryza*. Particular attention was paid to the role played by the most important classes of TEs (LTR RTs, LINEs, helitrons and other DNA transposable elements) in shaping these genomes and in contributing to genome size variation. In the case of four groups of TEs (the class 1 LTR-RTs and LINEs and the class 2 CACTA and *Mutator*-like elements) phylogenetic analyses were carried out.

## Results

**Figure 1:**
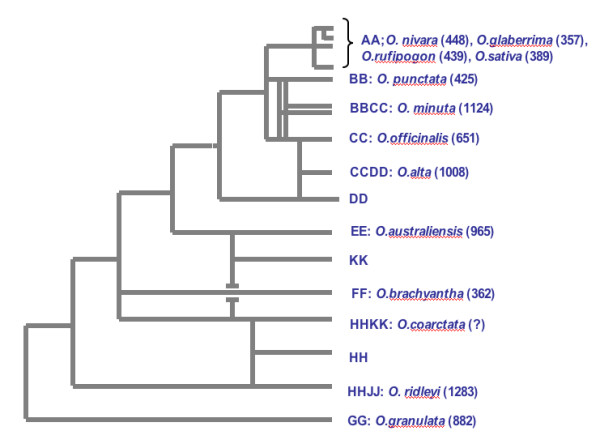
**Phylogeny of genus *Oryza***. Phylogenetic tree is a modified version of that proposed by Ge et al [53]. Figures between parentheses indicate the genome size in Million of nucleotides.

To better understand the repeat content of the genus *Oryza*, 12 random sheared libraries were constructed and sequenced from 12 vouchered accessions that represent the 10 genome types of *Oryza *(Figure 1). The total number of clones produced was 42,432, which roughly corresponded to 0.015× coverage of each genome. Mean insert size ranged from 2.5 kbp for *O. australiensis *[genome type:EE; genome size:965 Mbp] to 3.9 kbp for *O. rufipogon *[AA; 439 Mbp]. Cloned inserts were bi-directionally sequenced and after removing low quality data (sequences shorter than 50 bases and all those similar to plastid genomes), 72,245 high quality sequence reads were obtained for a total of 51.76 Mb of sequence (0.56% of the total size of all the genomes studied (Table 1)).

**Table 1:** Description of genomes investigated and libraries used

**Species**	**Genome type**	**Genome size (in Mbp)**	**Nr. of clones**	**Mean insert size (in Kb)**	**Genome fraction covered**	**Nr. of sequences**	**Mbp sequenced**	**% genome sequenced**
***O. nivara***	**AA**	448	1920	3.4	0.015	3103	2.04	0.46
***O. rufipogon***	**AA**	439	1920	3.9	0.017	2555	1.92	0.44
***O. glaberrima***	**AA**	357	1920	2.8	0.015	3494	2.68	0.75
***O. punctata***	**BB**	425	1920	3.0	0.014	3144	1.93	0.45
***O. officinalis***	**CC**	651	2688	3.3	0.014	4848	3.84	0.59
***O. minuta***	**BBCC**	1124	5376	2.9	0.014	9459	7.19	0.64
***O. alta***	**CCDD**	1008	4032	3.4	0.014	7538	5.58	0.55
***O. australiensis***	**EE**	965	4224	2.5	0.011	7359	4.68	0.48
***O. brachyantha***	**FF**	362	1920	3.3	0.018	3377	2.38	0.66
***O. granulata***	**GG**	882	4224	3.1	0.015	6688	4.45	0.50
***O. ridleyi***	**HHJJ**	1283	6144	3.5	0.017	11091	8.16	0.64
***O. coarctata***	**HHKK**	1283*	6144	3.0	0.014	9524	6.86	0.53

All the genome size estimates are from Ammiraju et al (2006) [4] except those of *O. glaberrima *and *O. minuta *which are from Martinez et al (1994) [54] and of *O. coarctata *which is a rough estimate because the real genome size of this species is unknown. We therefore used the value estimated for *O. ridleyi *[HHJJ; 1283 Mbp], which is also an allotetraploid species and shares the HH genome type with *O. coarctata*.

### Repeats abundance and distribution

Repetitive sequences were identified by similarity searches against databases of previously characterized repetitive elements, isolated from *O. sativa *and other *Oryza *species, including centromeric repeats, helitrons, SINEs, LINEs, MITEs, LTR-RTs, DNA transposable elements (DNA-TEs), ribosomal sequences and telomeric repeats (see Methods). All the major classes of repeats were represented throughout the genus (Table 2). The overall amount of repeats was quite variable in different species and ranged from the 25.04% of reads in *O. coarctata *[HHKK] to 66.42% of reads in *O. officinalis *[CC; 651 Mbp].

**Table 2:** Abundance of major classes of repeats in different *Oryza *genomes.

	*O. nivara *[AA; 448 Mbp]	*O. rufipogon *[AA; 439 Mbp	*O. glaberrima *[AA; 357 Mbp]	*O. punctata *[BB; 425 Mbp]	*O. officinalis *[CC; 651 Mbp	*O. minuta *[BBCC; 1124 Mbp]	*O. alta *[CCDD; 1008 Mbp]	*O. australiensis *[EE; 965 Mbp]	*O. brachyantha *[FF; 362 Mbp]	*O. granulata *[GG; 882 Mbp]	*O. ridleyi *[HHJJ; 1283 Mbp]	*O. coarctata *[HHKK]
Centromeric	0.64	0.59	2.29	4.39	3.84	3.66	6.22	0.91	2.04	0.30	0.25	0.16
Helitron	0.71	0.78	0.89	1.02	0.39	0.55	0.44	0.08	0.03	0.16	0.09	0.20
LINEs	1.10	1.17	1.09	0.83	0.47	0.68	0.81	0.86	0.36	0.28	0.39	0.58
MITEs	11.73	14.36	17.00	5.09	5.20	5.88	5.28	1.83	15.28	2.14	3.33	2.08
LTR-Retroelement (a)	3.87	4.38	4.49	5.38	6.60	5.26	7.95	5.25	2.34	4.65	8.75	2.73
Ty1-*copia*	2.32	1.92	2.78	3.47	3.96	5.10	5.35	9.84	2.49	4.74	4.59	7.44
Ty3-*gypsy*	23.27	19.69	13.31	20.42	31.31	24.27	18.81	35.59	7.88	45.60	19.38	7.74
Ribosomal	1.22	0.27	0.94	1.24	1.24	0.94	1.10	0.64	3.85	0.82	0.99	0.56
DNA-Transposon (b)	7.38	10.80	9.70	9.83	11.90	11.28	8.33	7.42	4.26	4.81	11.93	3.44
SINEs	0.35	0.63	0.49	0.03	0.21	0.23	0.25	0.01	0.18	0.07	0.11	0.00
Telomeric	0.06	0.16	0.00	0.10	0.27	0.12	0.16	0.26	0.00	0.01	0.08	0.04
Other (c)	0.68	0.94	0.97	0.22	1.03	0.38	2.84	0.10	0.03	0.07	0.21	0.07
Total	53.34	55.69	53.95	52.00	66.42	58.35	57.54	62.79	38.73	63.67	50.10	25.04

Occurrences are expressed as percentage of sequence reads having significant similarity out of the total.

(a) Refers to LTR-RTs that could not be classified as Ty3-*gypsy *or Ty1-*copia *(so non autonomous, tracts of LTRs from non autonomous etc.)

(b) Includes all the autonomous DNA elements; non autonomous ones such MITEs are excluded; helitrons are reported separately

(c) Unclassified repeats

In all the genomes studied the largest class of repeats consisted of LTR-RTs with amounts ranging from the 55% of reads in *O. granulata *[GG; 882 Mbp] to 12.7% of reads in *O. brachyantha *[FF; 362 Mbp]. DNA transposons, excluding MITEs and helitrons, appeared to be more abundant in *O. ridleyi *[HHJJ; 1283 Mbp] (11.93% of reads) and were depleted in *O. coarctata *[HHKK] (3.44% of reads).

The MITE sequences used as queries in similarity searches were isolated in *O. sativa *and not surprisingly they tag the highest value of hits in the three AA genome species: *O. glaberrima *(17% of reads), *O. nivara *(11.73% of reads) and *O. rufipogon *(14.36% of reads). The quantity of MITEs identified in the remaining genome types was less than 6% of reads except for the notable exception of the distantly related species *O. brachyantha *[FF; 362 Mbp] with 15.28% of reads. The lower than expected MITE content in the non-AA genomes species is likely due to the fact that MITEs are highly species specific and the repeat database used to identify MITEs was a curated data set from the *O. sativa *species only.

**Figure 2:**
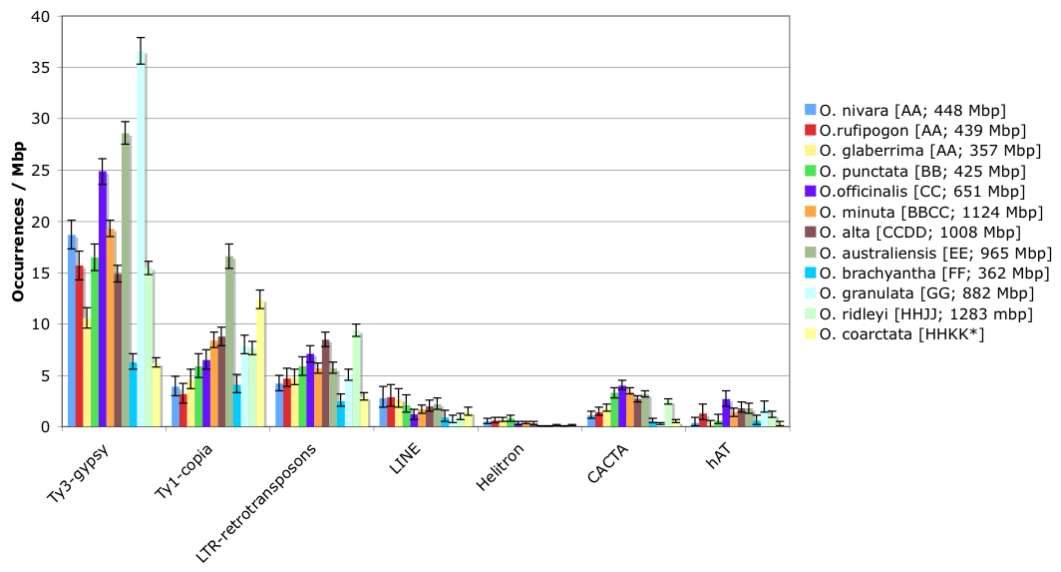
**Occurrence per single Mbp for the major classes of repeats in different *Oryza *genomes**. Y-axis: the estimated number of occurrences calculated using the equation from Hawkins et al (2006) [9] and normalized to 1 Mbp. The confidence intervals, displayed as error bars, were calculated assuming a Poisson distribution of repeats in the genome. The mean length used in calculation for different repeats were as follows: Ty3-*gypsy *elements: 12 Kbp; Ty1-*copia *elements: 5.5 Kbp; others LTR-RTs not classified (LTR-retrotransposons): 8.75 Kbp; LINEs: 3.5 kbp; helitrons (complete autonomous): 12.8 Kbp; CACTA: 15.2 Kbp; hAT: 3.6 Kbp *In this case all the calculations are based on a rough estimate of the genome size of this species: the real value is unknown, we therefore used the value estimated for *O. ridleyi *[HHJJ; 1283 Mbp], which is also an allotetraploid species and shares the HH genome type with *O. coarctata*.

Differences in TE size, host genome size and degrees of interspecific conservation make it particularly difficult to perform a detailed comparison based solely on the percentage of significantly similar sequences out of the total. A more rigorous approach was recently proposed by Hawkins et al. [9] where an equation was derived (see Methods) that takes into account TE mean size, host genome size, the minimum length needed in order to have a significant BLASTN hit, and the number of matches out of total reads available to estimate the absolute number of repeats in a given genome. We used this equation for all classes of repeats for which reliable estimates of the average length were available and then, in order to have comparable results, normalized the data to the single Mbp (Figure 2 and Additional file 1). Here we considered only the most represented and conserved families of elements: LTR-RTs, LINEs, helitrons, and the DNA-TEs – CACTA and *Mutator *like. It should however be noted how the use of average lengths for entire classes of elements could introduce a certain degree of uncertainty and so the following values should be considered as an approximation. Not surprisingly the LTR-RTs remained the major contributor to the repeat pool of each species. Species with the highest occurrence of LTR-RTs per Mbp were *O. granulata *[GG; 882 Mbp] (49.5) and *O. australiensis *[EE; 965 Mbp] (50.9) whereas the most depleted was *O*. *brachyanta *[FF; 362 Mbp] (12.9). In almost all cases, the Ty3-*gypsy *elements outnumbered Ty1-*copia *elements. This disproportion was particularly striking in the case of *O. nivara *[AA; 448 Mbp], *O. rufipogon *[AA; 439 Mbp] and *O. granulata *[GG; 882 Mbp] were the ratios Ty3-*gypsy *to Ty1-*copia *elements were 4.80, 4.91 and 4.6 respectively. The only exception to the overwhelming presence of Ty3-*gypsy *elements in comparison to Ty1-*copia *was found in *O. coarctata *[HHKK] where twice as many Ty1-*copia *elements (ratio of Ty3-*gypsy*/Ty1-*copia *= 0.5) were found.

Among other repeats, it is worth to note that CACTA elements seemed to be significantly more frequent in the BB (*O. punctata*; 3.3 elements per Mbp), CC (*O. officinalis*; 4.0 elements per Mbp), BBCC (*O. minuta*; 3.4. hits per Mbp), CCDD (*O. alta*; 2.7 hits per Mbp), EE (*O. australiensis*; 3.2 hits per Mbp) and HHJJ (*O. ridleyi*; 2.5 hits per Mbp) genomes as compared with all 3 AA genomes (*O. glaberrima*, *O. rufipogon*, *O. nivara*) where the number of hits was less than 2 per Mbp. In almost all the genomes the frequency of CACTA elements was greater than the hAT elements, with the exception of *O. brachyantha *[FF; 362 Mbp] (CACTA = 0.5 hits/Mbp; hAT 0.6 hits/Mbp) and *O. granulata *[GG; 882 Mbp] in which hAT elements outnumber the CACTA elements 6 to 1 (CACTA = 0.3 hits/Mb; hAT = 1.9/Mbp). The distribution of LINEs was higher in the AA genomes where it was greater than 2.5 hits per Mbp and very low in *O. granulata *[GG; 882 Mbp] with only 0.7 hits per Mbp.

The significant variance of LTR-RT representation presented above prompted us to perform a more in depth analysis of the distribution of the different families in each genome. To identify the primary LTR-RT families responsible for this variance and possibly link them to the genome size variation in each host species, all sequences previously identified as being similar to LTR-RTs were screened a second time against a reduced database containing representatives of twenty-six of the most abundant LTR-RT families isolated in *Oryza *genus (11 Ty1-*copia *and 15 Ty3-*gypsy*) (Table 3). In all species the overall majority of sequences already annotated as LTR-RTs could be easily classified according to these parameters (Table 3). Elements related to the most abundant families of LTR-RTs isolated in *O. sativa *[AA; 389 Mbp] were identified in all the *Oryza *genome types considered with very few exceptions. This indicated that the complement of LTR-RTs previously identified in rice is shared throughout the *Oryza *genus. However, as shown in Table 3, different LTR-RTs had different fates in different species.

**Table 3:** Abundance of major LTR-RTs families in different species of the genus Oryza

	*O. nivara*	*O. rufipogon*	*O. glaberrima*	*O. punctata*	*O. officinalis*	*O. minuta*	*O. alta*	*O. australiensis*	*O. brachyantha*	*O. granulata*	*O. coarctata*	*O. ridleyi*
COPIA1	0.35	0.59	0.60	0.57	0.87	0.82	0.57	1.64	0.18	0.69	0.81	0.63
COPIA2	0.19	0.39	0.26	0.19	0.27	0.35	0.54	0.45	0.12	0.70	0.21	0.27
COPIA3	0.13	0.00	0.00	0.19	0.00	0.37	0.32	0.43	0.15	0.07	0.18	0.31
COPIO	0.42	0.31	0.31	0.92	0.60	0.85	0.62	0.48	0.65	0.84	0.71	0.96
Hopscotch	0.03	0.08	0.06	0.03	0.06	0.10	0.05	0.04	0.00	0.10	0.48	0.14
OSTONOR1	0.16	0.04	0.11	0.32	0.37	0.51	0.11	0.79	0.24	0.22	0.02	0.14
RETROFIT4	0.10	0.00	0.06	0.06	0.06	0.06	0.09	0.07	0.06	0.04	0.35	0.06
RIRE1	0.06	0.00	0.00	0.16	0.10	0.60	0.61	4.38	0.03	0.49	0.23	0.46
RIRE5	0.26	0.16	0.26	0.06	0.17	0.18	0.42	0.15	0.06	0.01	0.05	0.15
SC-1	0.06	0.04	0.11	0.10	0.17	0.26	0.46	0.71	0.09	0.34	0.04	0.37
SZ-6	0.03	0.00	0.03	0.03	0.04	0.08	0.17	0.08	0.09	0.12	2.75	0.14
**ATALANTYS**	1.87	1.49	1.77	6.36	10.15	8.70	7.32	10.98	0.36	7.28	4.08	7.62
**BAJIE**	0.26	0.39	0.66	0.54	0.74	0.49	0.45	0.15	0.00	0.04	0.04	0.22
**RIREX (Dasheng)**	0.55	1.06	0.20	1.02	1.49	1.08	0.74	1.24	0.03	1.26	0.05	0.53
**GYPSY1**	0.48	0.23	0.40	0.22	0.27	0.29	0.16	1.18	0.18	0.30	0.18	0.05
**RETRO2**	1.68	1.10	0.26	1.08	3.03	1.31	0.72	0.54	0.06	0.96	0.16	1.97
**RETROSAT2**	1.64	0.78	0.52	0.54	1.65	1.63	0.86	2.74	4.12	1.84	0.05	0.38
**RETROSAT3**	0.68	0.98	0.66	0.70	1.69	1.62	1.01	2.81	2.13	1.66	0.05	0.37
**RETROSAT4**	1.29	0.78	0.54	1.02	2.76	0.88	0.70	0.84	0.03	1.75	0.22	1.12
**RIRE2**	1.61	1.02	0.74	1.69	4.43	2.06	1.70	8.56	0.24	21.50	0.41	2.42
**RIRE3**	3.42	3.37	1.09	0.25	0.58	0.43	0.17	0.76	0.06	0.60	0.18	0.41
**RIRE8**	5.90	5.21	2.55	1.18	0.78	1.08	1.49	0.86	0.24	2.59	0.29	0.31
**SZ-22**	0.19	0.27	0.11	0.32	0.33	0.42	0.19	0.16	0.00	0.06	0.00	0.07
**SZ-42**	0.23	0.39	0.26	0.06	0.19	0.24	0.21	0.18	0.06	1.11	0.04	0.61
**SZ-50**	0.77	0.47	0.49	2.19	0.06	0.85	0.07	0.03	0.00	0.36	0.12	0.06
**SZ-7**	1.03	0.90	1.35	0.57	1.38	1.00	0.92	0.31	0.03	1.05	0.31	0.51
UNC	2.55	3.29	3.18	3.85	4.76	3.41	5.64	3.66	1.63	2.57	1.23	3.21
NHF	1.32	1.14	1.32	1.53	1.84	1.85	2.31	1.59	0.71	2.08	1.50	5.55
Other Families	2.19	1.54	2.57	3.69	3.03	3.11	3.46	4.86	1.18	4.35	3.15	3.70

Figures represent the percentage of significant hits out of the total number of reads for each species. Ty3-*gyspy *family names are in bold; Ty1-*copia *ones are not. UNC refers to elements that in RepBase were classified as LTR-RT but without any other detail (i.e. Ty1-*copia *or Ty3-*gypsy*); NHF refers to elements that are LTR-RTs related but do not have significant similarity with the major families isolated in the genus *Oryza*. "Other families" indicates significant hits with other *Oryza sativa *LTR-RTs elements than those belonging to the families analyzed here.

A paradigmatic case was that of two Ty3-*gypsy *families *Atlantys *and *RIRE2 *(that is related to the element *Wallabi *[10]). *Atlantys *was the most abundant element in 7 of the 10 *Oyrza *genome types (*O. punctata *[BB; 425 Mbp], 6.36% of total reads; *O. officinalis *[CC; 651 Mbp], 10.15% of total reads; *O. minuta *[BBCC; 1124 Mbp], 8.70% of total reads; *O. alta *[CCDD; 1008 Mbp], 7.32% of total reads; *O. australiensis *[EE; 965 Mbp], 10.98% of total reads; *O. coarctata *[HHKK], 4.08% of total reads and *O. ridleyi *[HHJJ; 1283 Mbp], 7.62% of total reads). It was also the second most abundant element in *O. granulata *[GG; 882 Mbp], 7.28% of total reads. However in the AA and FF genomes, it did not expand significantly in comparison to the other elements. *RIRE2 *related elements (i.e. *Wallabi *[10] in *O. australiensis*) expanded significantly in *O. australiensis *[EE; 965 Mbp], 8.56% of the total reads and in *O. granulata *[GG; 882 Mbp], 21.5% of total reads.

It is also worth to note that the *RIRE8 *Ty3-*gypsy *element is more widespread in the AA genomes (5.90% and 5.21% of total reads in *O. nivara *and *O. rufipogon*, respectively) than in the remaining genome types.

The *O. minuta *[BBCC; 1124 Mbp] genome represents the only polyploid genome for which a comparison with both the original genome counterparts BB (*O. punctata*) and CC (*O. officinalis*) is possible. All LTR-RT families identified in *O. minuta *[BBCC: 1124 Mbp] were found in at least one of the diploid counterparts and there were only few cases where an element was significantly more represented in the polyploid genome than in the two diploid counterparts. The most convincing example was that of the Ty1-*copia *element *RIRE1 *(0.16%, 0.1% and 0.6% of total reads in *O. punctata*, *O. officinalis *and *O. minuta *respectively).

For all the genome types it was possible to identify a fraction of LTR-RT related sequences that did not show any significant similarity with the major families isolated from *O. sativa*. The amount of this fraction expressed as percentage out of the total number of sequence reads ranged from 0.71% in *O. brachyantha *[FF; 362 Mbp] to 5.6% in *O. ridleyi *[HHJJ; 1283 Mbp] and represented the most species specific sets of these elements (or the most diverged from the *O. sativa *retroelement pool).

Our strategy to rely on similarity searches in order to identify the repetitive fraction of a genome has the obvious pitfall that, if a repeat is not present in the repeat database it will not be detected. To check for this possibility we adopted a different approach to screen all sequence reads that did not show any significant hits with already characterized repeats. Sequences were clustered using the program Blastclust and the results are shown in Table 4. Only a few clusters containing more than 3 sequences were isolated in all the species with the exception of the AA and BB genomes in which no clusters were identified. The largest cluster identified was found in *O. ridleyi *[HHJJ; 1283 Mbp] and contained 69 sequences out of 11,091 (0.62%). The sequences present in these clusters very likely represent previously uncharacterized repeats. However as the figures for *O. ridleyi *[HHJJ; 1283 Mbp] demonstrate, they do not constitute a significant portion of the genomes in which they were isolated.

**Table 4:** Clustering analysis

A	B	C	D
*O. glaberrima *[AA; 357 Mbp]	1610	0	-
*O. rufipogon *[AA; 439 Mbp]	1133	0	-
*O. nivara *[AA; 448 Mbp]	1448	0	-
*O. punctata *[BB; 425 Mbp]	1508	0	-
*O. officinalis *[CC; 651 Mbp]	1626	4	5
*O. minuta *[BBCC; 1124 Mbp]	3940	3	10
*O. alta *[CCDD; 1008 Mbp]	3198	19	24
*O. australiensis *[EE; 965 Mbp]	2738	16	11
*O. brachyantha *[FF; 362 Mbp]	2069	4	15
*O. granulata *[GG: 882 Mbp]	2429	9	7
*O. coarctata *[HHKK]	7143	8	21
*O. ridleyi *[HHJJ; 1283 Mbp]	5546	65	69

Results of the clustering analysis carried out on the sequence reads that did not show any significant hits with previously annotated repeats. B: Number of sequence reads. C: Number of clusters containing more than 3 sequences. D: Number of sequences included in the largest cluster.

### Phylogenetic analysis of repeats

To establish the phylogeneic relationships among the most prominent superfamilies of TEs in *Oryza*, we used amino acid sequences of the most conserved domain tracts (see Methods) of Ty1-*copia *and Ty3-*gypsy *LTR-RTs, LINEs and CACTA and *Mutator*-like DNA-TEs to build neighbor-joining trees.

**Figure 3:**
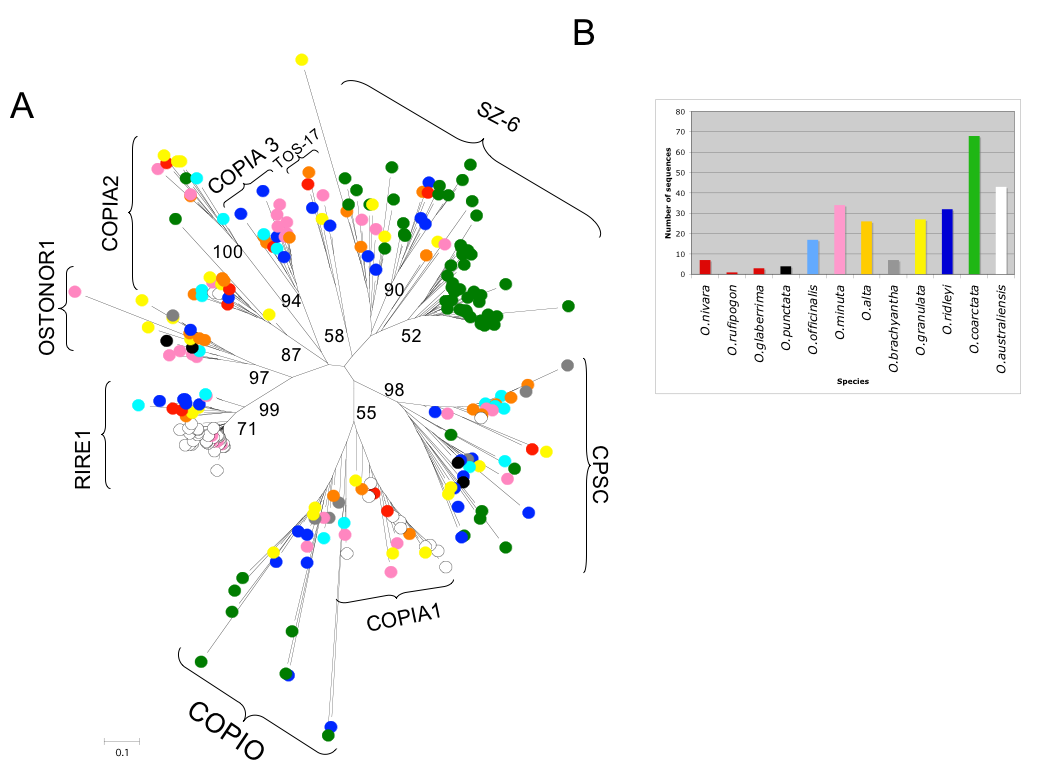
**Phylogenetic analysis of Ty1-*copia *retroelements**. A) 269 sequences similar to the Ty1-*copia *reverse transcriptase domain were used to build a phylogenetic tree using the neighbor-joining method. Bootstrap values were calculated for 1000 replicates; only those with values greater than 50 are proposed B) distribution of the domains isolated in different species. Bar colors are the same of those used in the circles marking, on the neighbor-joining tree sequences from different species.

For Ty1-*copia *elements (Figure 3) it was possible to retrieve 269 reverse transcriptase (RT) sequences out of the 72,245 available sequences. These sequences were resolved into at least eight lineages as representatives of the major Ty1-*copia *retroelements isolated. The amount of sequences in each lineage reflected the abundance of that family in the genome. Furthermore the most abundant lineages contained representatives of all the different species and there was not a single species-specific lineage with the partial exception of *RIRE1 *in *O. australiensis *[EE; 965 Mbp]. This lineage, also including a few sequences from *O. minuta *[BBCC; 1124 Mbp], was separated from the others and it was possible to see how the branch lengths are short suggesting that amplification of this element took place after speciation (with the possible exception represented by the few *O. minuta RIRE1 *related sequences).

**Figure 4:**
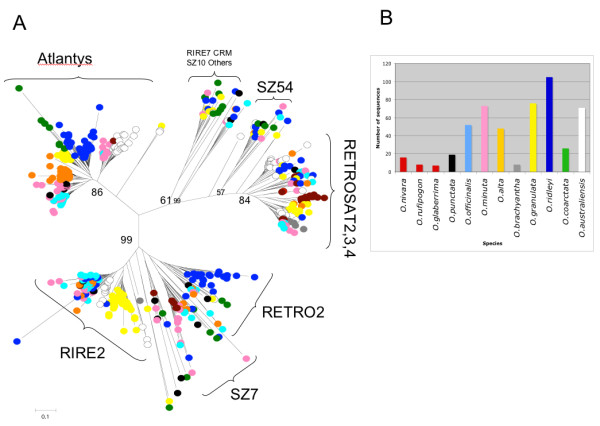
**Phylogenetic analysis of Ty3-*gypsy *retroelements**. A) 508 sequences similar to the Ty3-*gypsy *reverse transcriptase domain were used to build a phylogenetic tree using the neighbor-joining method. Bootstrap values were calculated for 1000 replicates; only those with values greater than 50 are proposed B) distribution of the domains isolated in different species. Bar colors are the same of those used in the circles marking, on the neighbor-joining tree sequences from different species.

RT-like sequences from Ty3-*gypsy *elements (508 elements) were isolated and used to build a phylogenetic tree (Figure 4). The complete set is less heterogeneous than the Ty1-*copia *set, however all the major families are represented. In the case of *RIRE2 *and *Atlantys*, the two most abundant Ty3-*gypsy *families throughout the genus, all the species were included in the correspondent lineages indicating that their presence in the genus predates its speciation. However, considering the different species in which the major amplification events of elements belonging to these two families took place, it was possible to note how the elements isolated in different species usually do not mix together in the internal lineages suggesting that their amplification has happened after speciation.

**Figure 5:**
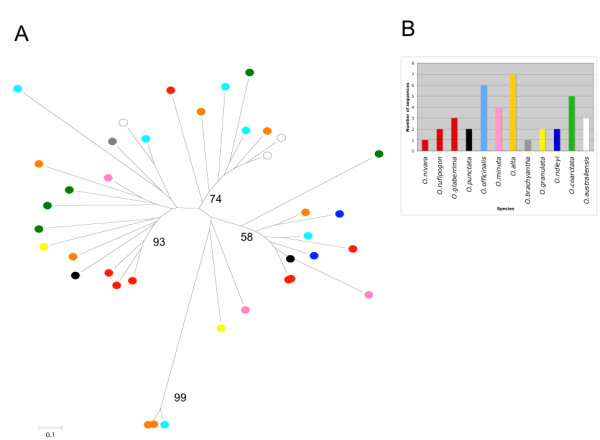
**Phylogenetic analysis of LINE retroelements**. A) 38 sequences similar to the LINE reverse transcriptase domain were used to build a phylogenetic tree using the neighbor-joining method. Bootstrap values were calculated for 1000 replicates; only those with values greater than 50 are proposed B) distribution of the domains isolated in different species. Bar colors are the same of those used in the circles marking, on the neighbor-joining tree sequences from different species.

LINEs are usually not very abundant in plants as compared to LTR-RTs with some exceptions such as the *del2 *element in *Lilium speciosum *[30]. The genus *Oryza *in this sense appears to follow this general rule. This trend is reflected in the number of LINE reverse transcriptase like sequences [38] isolated from our set. Twenty-two of them cluster in 4 major bootstrap supported lineages and many are characterized by very long branches suggestive of an ancient origin of these retroelements in the genus (Figure 5). These findings suggest that even in the case of the *Oryza *genus, LINEs exhibit, as in other plants, high sequence divergence and extreme heterogeneity [31-33].

**Figure 6:**
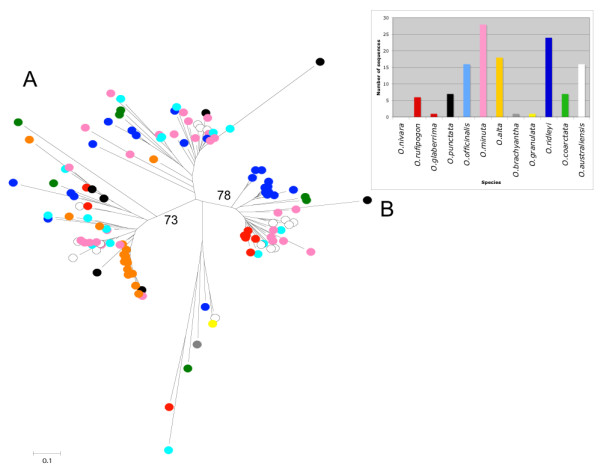
**Phylogenetic analysis of CACTA transposable elements**. A) 125 sequences similar to the CACTA transposase domain were used to build a phylogenetic tree using the neighbor-joining method. Bootstrap values were calculated for 1000 replicates; only those with values greater than 50 are proposed B) distribution of the domains isolated in different species. Bar colors are the same of those used in the circles marking, on the neighbor-joining tree sequences from different species.

**Figure 7:**
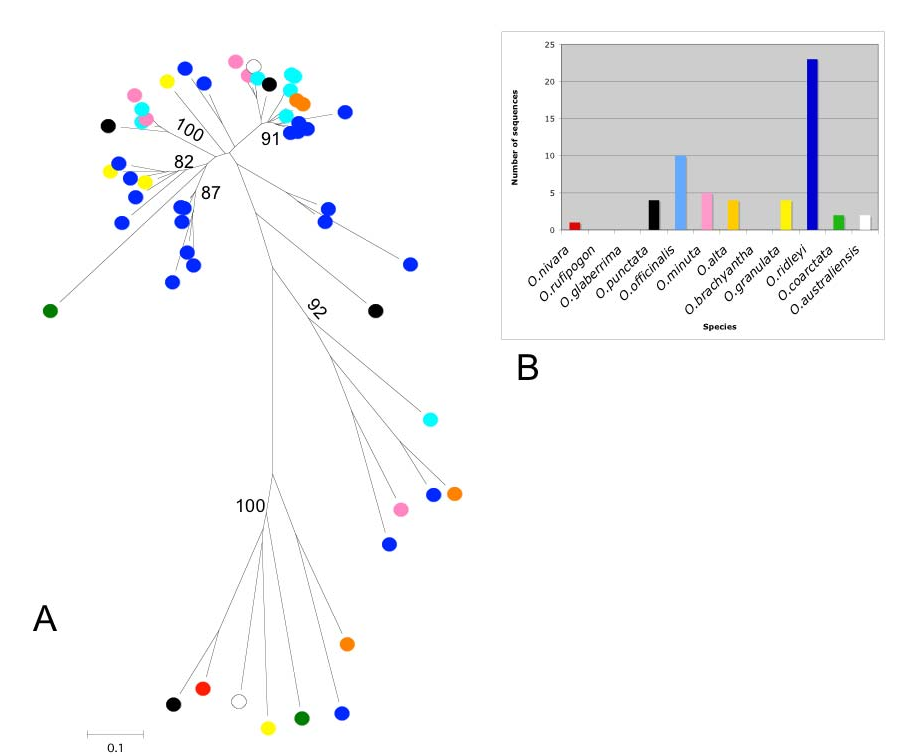
**Phylogenetic analysis of *Mutator*-like transposable elements**. A) 55 sequences similar to the *Mutator*-like transposase domain were used to build a phylogenetic tree using the neighbor-joining method. Bootstrap values were calculated for 1000 replicates; only those with values greater than 50 are proposed B) distribution of the domains isolated in different species. Bar colors are the same of those used in the circles marking, on the neighbor-joining tree sequences from different species.

The two major groups of DNA TEs, CACTA and *Mutator*-like were phylogenetically investigated using tracts of their transposase coding domains. In the case of CACTA elements, we identified 125 transposase-like sequences, whereas 55 were isolated for *Mutator*-like elements. For both classes of TE elements, it was not possible to identify species specific lineages in the corresponding Neighbor Joining trees (Figures 6 and 7). The only exception was represented by a large lineage including 14 of the 18 CACTA transposase domains isolated in *O. alta *[CCDD; 1008 Mbp]. However this lineage lacks consistent bootstrap support.

## Discussion

Genome survey sequences from 12 random sheared genomic libraries representative of the 10 genome types of the genus *Oryza *were generated and analyzed to obtain an unbiased sampling of the repeat content of the genus as a whole. Our results showed that the genomic composition of the *Oryza *species is not exceptional when compared to other plant species in that the repetitive fraction consistently makes up a significant portion of each of the 10 genome types.

Among TEs, the major class of repeats in *Oryza *was represented by LTR-RTs whose absolute occurrence was estimated to be in the order of thousands of copies for each species. Detailed analysis of the major *O. sativa *LTR-RT families enabled us to identify the elements responsible for the major differences in genome size variation across the genus *Oryza*. For example, LTR-RTs related to *RIRE2 *represent more than 8.5% and 21% of all the sequences obtained for *O. australiensis *[EE; 965 Mbp] and *O. granulata *[GG; 882 Mbp] respectively. This is consistent with previous studies which showed that the proliferation of 3 family members (*RIRE1*, the *RIRE2 *related element *Wallabi *and *Kangaroo*) in EE genomes [10] and a single family (*Gran3*) in the GG genome [Jetty et al., 2007 accepted with revisions] accounts for nearly 50% and 25% increases in their genome sizes respectively. Another family that has played a significantly similar role in genome size variation is the Ty3-*gypsy *element *Atlantys*. The *Atlantys *family has been surprisingly overlooked by previous studies and with its amplification has impacted not only the *O. granulata *and *O. australiensis *genomes but also the BB, CC, BBCC and CCDD genomes. The events leading to the explosive proliferation of both the *RIRE2 *and *Atlantys *families appear to have taken place after each speciation event.

The consequences of TE proliferation subsequent to speciation on genome size variation is reminiscent of *Zea mays *in which five LTR-RT families represent nearly 25% of the genome [34] and of the species *Gossypium herabaceum *and *G. exiguum *where a group of Ty3-*gypsy *elements, named *GORGE*-*3*, has extensively proliferated in comparison to other elements and constitutes a significant portion of each genome [9]. In contrast, no single LTR-RT family was found to constitute significant portions of the AA genome *Oryza *species with the only exception being represented by the Ty3-*gypsy *element *RIRE8*. The finding that, apart from the polyploids, the largest genomes in the *Oryza *(*O. australiensis *and *O. granulata*) are those that have seen the "explosive" proliferation of one or two LTR-RT families, once again identifies the pivotal role of this class of TEs in the dynamics of genome size variation.

The differential proliferation of LTR-RTs in the *Oryza *genus could be explained by taking into account the effects of varying genomic backgrounds on element life cycles, different rates of LTR-RT removal by unequal recombination and/or illegitimate recombination [8,15,16] and inherently different retrotranspositional potentials of various element families [35]. However no single convincing explanation of this phenomenon has so far been advanced for other plant species in which similar events have been described [9,10].

Here we have demonstrated that the pool of LTR-RTs in the *Oryza *is substantially conserved throughout the genus because in almost all cases relatives of the LTR-RT families identified in *O. sativa *[AA] could also be found in the other *Oryza *species. We therefore conclude that its existence predates the speciation events that originated the genus as we know. This finding is well supported by our phylogenetic analyses and it is in accordance with the results of previous studies analyzing the Ty1-*copia *elements phylogeny and distribution across different genera of the Gramineae family [36,37].

Besides the identification of different amounts of elements from the same LTR-RT families, all the genomes studied, except *O. coarctata*, showed the same quantitative relationship between Ty3-*gypsy *and Ty1-*copia *elements where the Ty3-*gypsy *elements constantly outnumber Ty1-*copia *elements, even if the ratio between the two elements varied (from more than 4.6 in *O. nivara *[AA; 448 Mbp], *O. rufipogon *[AA; 439 Mbp] and *O. granulata *[GG; 882 Mbp] to 1.52 in *O. brachyantha *[FF; 362 Mbp]). This data is completely consistent with previous analysis of the cultivated *japonica *and *indica *genomes [25,38-41] and can now be extended to the genus *Oryza*, with the notable exception of *O. coarctata *[HHKK] where we found a ratio close to 0.5 between the Ty3-*gypsy *and Ty1-*copia *elements.

An even more striking difference between the *O. coarctata *[HHKK] genome and the other *Oryza *spp genomes is that it was found to contain the least amount repetitive elements despite it being polyploid and its predicted large genome size. It should be noted that we have not been able to obtain live plants or tissues in the U.S. in order to determine an accurate genome size measurement by flow cytometry due to quarantine restrictions. To rule out the possibility that our similarity searches could not detect highly diverged elements we analyzed all the sequences to try to identify repeats *de novo *through a clustering approach. In this case no significant de novo clusters were obtained in *O. coarctata*. These findings rule out the existence of a single recently originated family of repeats, highly species specific and constituting a significant portion of the genome. However, the results do not discount the possibility that repeats missed by previous searches in *O. coarctata*, if present, belong to several different families, possibly diverged (and so ancient). All evidence combined supports the old taxonomic view that places *O. coarctata *in the genus *Porteresia *[42] and not *Oryza*. However more in depth and accurate analyses are needed in order to ascertain the dynamics acting towards this apparently repeat depletion in *O. coarctata*.

The evolutionary events leading to the present day *Oryza *genus spanned at least 9 million years from *O. sativa *[AA; 389 Mbp] to the the most basal species *O. granulata *[GG; 882 Mbp] [43]. Similar to what was observed in *O. coarctata *[HHKK], the use of repeat collections mainly based on elements isolated in *O. sativa *[AA;389 Mbp] to screen other species of the genus raises some concerns about how nucleotide divergence can impact estimates of LTR-RTs abundance. With the exceptions of *O. coarctata *(discussed above) and *O. brachyantha *[FF; 362 Mbp] (in which the depletion of repeats can be easily explained taking into account the small size of this genome), our work demonstrated that the amount of LTR-RTs isolated does not decrease substantially when our analysis moved from the AA genome types to the most distant ones. The evidence has been indirectly confirmed by the clustering analysis that did not revealed the existence any significantly repeat family previously missed. This, in turn, demonstrates how similarity searches of LTR-RT families have a good level of "portability" throughout the genus giving substantially reliable and reasonably exhaustive results, if the proper settings are used.

A completely different scenario could be described for TEs lacking conserved domains and having higher species sequence specificity such as MITEs and SINEs. In this case the evolutionary distance from AA genomes consistently results in a steady drop in the amount TEs identified (with the significant exception of MITEs in *O. brachyantha*) prompting the need of *de novo *and *ad hoc *identification tools based on structural features beside the nucleotidic sequence.

## Conclusion

Here we report the results of a comprehensive analysis of the abundance and relative distribution of major TE classes across twelve species of the genus *Oryza*.

We have demonstrated how the LTR-RT complement in of these species is ancient and conserved throughout the genus, and has attained different retrotranpositional success in different species.

We also identified two LTR-RT families (*RIRE2 *and *Atlantys*) that are responsible for a significant portion of genome size variation in the genus and we demonstrated how their massive increase in copy number is recent.

## Methods

### Plant material (put in genome designations)

Total genomic DNA was isolated from young leaf tissue from the following *Oryza *species: *O. brachyantha *[FF; 362 Mbp] (Acc# 101232), *O. alta *[CCDD;1008 Mbp] (Acc# 105143), *O. officinalis *[CC; 651 Mbp] (Acc# 100896), *O. ridleyi *[HHJJ; 1283 mbp](Acc# 100821), *O. punctata *[BB; 425 Mbp](Acc# 105690), *O. coarctata *[HHKK](Acc# 104502), *O. minuta *[BBCC; 1124 mbp](Acc# 101141), *O. granulata *[GG; 882 Mbp](Acc# 102118), *O. glaberrima *[AA; 357 Mbp](Acc# 96717), *O. rufipogon *[AA; 439 mbp] (Acc# 105491), *O. nivara *[AA; 448 Mbp] (Acc# 100897) and *O. australiensis *[EE; 965 Mbp] (Acc# 100882). Plant materials were grown at the International Rice Research Institute, Philippines.

### Library construction and DNA sequencing

Genomic DNA was extracted using CTAB [44]. DNA was sheared using the Hydroshear (GeneMachines) to produce 2–5 kb fragments. DNA fragments were end-repaired using Epicientre's End-it Repair kit and size selected on 0.6% agarose gels. DNA fragments in the size range of 2–5 kb were cut out of the gel, eluted using a QIAEX II Gel extraction kit and ligated to linearized pBluescriptII KS+ at 16°C overnight. Ligation products (1 uL) were transformed into DH10B electrocompetent cells (Invitrogen). Shotgun plasmids were sequenced bi-directionally on ABI 3730XL DNA sequencers (ABI) using standard protocols. Sequence data was extracted using ABI sequence analysis software and base called using Phred [45]. Vector screening and low quality sequence removal was done using the program Lucy [46].

### Similarity searches

Sequences were used as queries in similarity searches against different repeat databases: TIGR rice repeats version 3.1 [47], Repbase version 11_09 [48] and a proprietary collection of retroelements isolated in *Oryza sativa *and other *Oryza *species. Searches were carried out using the BLASTN algorithm [49] run under relaxed settings (-q -2 -r 3) in order to accommodate the divergence between species searched from the major source of repeats in our database (*O. sativa*). Only hits having an E value equal or lower to 1e-10 were used. For all sequences that did not result in any significant hits, a second round of searches was carried out using the algorithm BLASTX against the non-redundant division of GenBank. Under these conditions only hits having an E value equal or lower to 1e-5 were parsed. To estimate the number of significantly similar hits present in the entire genome of each of the 12 *Oryza *species, we used this equation: n = (Xobs/N)*(G/(L-2m+e) that is a slight modification of that proposed by Hawkins et al. [9], where "Xobs" is the observed number of copies, "N" is the total number of sequence reads, "n" is number of targets in the genome, "L" is length of target sequence, "m" is estimated minimum length required to identify a sequence in a BLAST search (we used the extremely conservative value of 100 bp), "e" is number of bp sequenced from each insert and "G" is genome size. Published sequences for various repetitive elements were used to estimate "L".

A third round of similarity searches was performed using only representative elements from the LTR-RT families listed in Table 3.

All sequences that were not classified as repeats were used in a cluster analysis using "Blastclust" [50]. The program was used with the following settings, L 0.51 S 80, meaning that all sequences sharing at least 80% similarity over at least 51% of their length were included in the same cluster. Only clusters containing 3 or more hits were considered in analyzed further.

### Phylogenetic analysis

Sequences homologous to the Reverse Transcriptase domains of Ty1-*copia *([GenBank: CAD40165.2], 92 AA from residues 781 to 872), Ty3-*gypsy *([GenBank: CAH66235.1],162 AA from residues 471 to 632) and LINEs (122 residues from RT domain of element OSLINE1_5 in Repbase [48]) and sequences homologous to transposase domains of CACTA ([GenBank: DAA02108.1], 100 AA from residue 1 to 100) and *Mutator*-like elements ([GenBank: AAM94534.1], 238 AA from residues 361 to 598), were identified in the random sheared libraries through tBLASTN searches [49]. The corresponding amino acid domains were retrieved and aligned using Muscle [51]. Neighbor-joining trees were produced and edited using the program MEGA version 3 [52].

### Accession numbers

Sequences for this paper were submitted to the GSS division of GenBank under the following accessions numbers: [GenBank: EI028463–EI035999] (*O. alta*), [GenBank: EI36000–EI043358] (*O. australiensis*), [GenBank: EI043359–EI046735] (*O. brachyantha*), [GenBank: EI46736–EI56259] (*O. coarctata*), [GenBank: EI056260–EI59753] (*O. glaberrima*), [GenBank: EI059754–EI066441] (*O. granulata*), [GenBank: EI066442–EI075900] (*O. minuta*), [GenBank: EI-075901–EI079003] (*O. nivara*), [GenBank: EI079004–EI083851] (*O. officinalis*), [GenBank: EI083852–EI86995] (*O. punctata*), [GenBank: EI086996–EI098086] (*O. ridleyi*), [GenBank: EI098087–EI100641] (*O. rufipogon*).

## Authors' contributions

AZ carried out the analysis, wrote the manuscript and participated in the design of this study. AS: contributed to the phylogenetic analysis and to the data parsing. JT: constructed the genomic libraries. YY, HK, KC and DK participated in the library construction and the production of sequencing data. RAW contributed to the design of the study, supervised the experimental steps and contributed to the preparation and editing of the manuscript. All authors read and approved the final manuscript.

## Supplementary Material

Additional file 1 Occurrences per single Mbp for the major classes of repeats in different *Oryza *genomes. A) The absolute number of significant hits to the *Oryza *repeat data bases. C: Estimated number of occurrences calculated using the equation from Hawkins et al (2006) [9]. E: The number normalized to 1 Mbp. Columns B, D and F are the confidence intervals calculated assuming a Poisson distribution of repeats in the genome, calculated for values in columns A, C and E respectively. The mean length used for different repeats were as follows: Ty3-*gypsy *elements: 12 Kbp; Ty1-*copia *elements: 5.5 Kbp; others LTR-RTs not classified: 8.75 Kbp; LINEs: 3.5 kbp; Helitrons (complete autonomous): 12.8 Kbp; CACTA: 15.2 Kbp; hAT: 3.6 Kbp. *In this case all the calculations are based on a rough estimate of the genome size of this species: the real value is unknown; we therefore used the value estimated for *O. ridleyi *[HHJJ; 1283 Mbp], which is also an allotetraploid species and shares the HH genome type with *O. coarctata*. Click here for file
