# Supplementary material for: Transposable element distribution, abundance and role in genome size variation in the genus Oryza
Source: BMC Evol Biol. 2007 Aug 29;7:152. doi: 10.1186/1471-2148-7-152 (PMC2041954; doi:10.1186/1471-2148-7-152)
Supplement: Additional file 1 — Occurrences per single Mbp for the major classes of repeats in different Oryza genomes. A) The absolute number of significant hits to the Oryza repeat data bases. C: Estimated number of occurrences calculated using the equation from Hawkins et al (2006) [9]. E: The number normalized to 1 Mbp. Columns B, D and F are the confidence intervals calculated assuming a Poisson distribution of repeats in the genome, calculated for values in columns A, C and E respectively. The mean length used for different repeats were as follows: Ty3-gypsy elements: 12 Kbp; Ty1-copia elements: 5.5 Kbp; others LTR-RTs not classified: 8.75 Kbp; LINEs: 3.5 kbp; Helitrons (complete autonomous): 12.8 Kbp; CACTA: 15.2 Kbp; hAT: 3.6 Kbp. *In this case all the calculations are based on a rough estimate of the genome size of this species: the real value is unknown; we therefore used the value estimated for O. ridleyi [HHJJ; 1283 Mbp], which is also an allotetraploid species and shares the HH genome type with O. coarctata. [file 1471-2148-7-152-S1.doc]

| ‘ | A | B | C | D | E | F |
| --- | --- | --- | --- | --- | --- | --- |
| ***O. nivara* [AA; 448 Mbp]** |  |  |  |  |  |  |
| *ty-3*-*gypsy* | **722** | 670.3 - 776.6 | **8368** | 7769 - 9001 | **18.7** | 17.3 - 20.1 |
| *ty-1*-*copia* | **72** | 56.3 - 90.7 | **1745** | 1365 - 2198 | **3.9** | 3 - 4.9 |
| retrotransposon | **120** | 99.5 - 143.5 | **1882** | 1560 - 2250 | **4.2** | 3.5 - 5 |
| LINE | **34** | 23.5 - 47.5 | **1241** | 859 - 1734 | **2.8** | 1.9 - 3.9 |
| Helitron | **22** | 13.8 - 33.3 | **240** | 150 - 363 | **0.5** | 0.3 - 0.8 |
| CACTA | **54** | 40.6 - 70.5 | **498** | 374 - 650 | **1.1** | 0.8 - 1.5 |
| hAT | **5** | 1.6 - 11.7 | **178** | 58 - 415 | **0.4** | 0.1 - 0.9 |
|  |  |  |  |  |  |  |
| ***O. rufipogon*[AA; 439 Mbp]** | |  |  |  |  |  |
| *ty-3*-*gypsy* | **503** | 460 - 548.9 | **6886** | 6297 - 7515 | **15.7** | 14.3 - 17.1 |
| *ty-1*-*copia* | **49** | 36.3 - 64.8 | **1391** | 1029 - 1839 | **3.2** | 2.3 - 4.2 |
| retrotransposon | **112** | 92.2 - 134.8 | **2069** | 1704 - 2490 | **4.7** | 3.9 - 5.7 |
| LINE | **30** | 20.2 - 42.8 | **1272** | 859 - 1816 | **2.9** | 2 - 4.1 |
| Helitron | **20** | 12.2 - 30.9 | **257** | 157 - 398 | **0.6** | 0.4 - 0.9 |
| CACTA | **58** | 44 - 75 | **633** | 480 - 818 | **1.4** | 1.1 - 1.9 |
| hAT | **14** | 7.7 - 23.5 | **579** | 317 - 972 | **1.3** | 0.7 - 2.2 |
|  |  |  |  |  |  |  |
| ***O. glaberrima*[AA; 357 Mbp]** | |  |  |  |  |  |
| *ty-3*-*gypsy* | **465** | 423.7 - 509.2 | **3781** | 3445 - 4140 | **10.6** | 9.6 - 11.6 |
| *ty-1*-*copia* | **97** | 78.7 - 118.3 | **1634** | 1325 - 1993 | **4.6** | 3.7 - 5.6 |
| retrotransposon | **157** | 133.4 - 183.6 | **1722** | 1463 - 2013 | **4.8** | 4.1 - 5.6 |
| LINE | **38** | 26.9 - 52.2 | **955** | 676 - 1310 | **2.7** | 1.9 - 3.7 |
| Helitron | **31** | 21.1 - 44 | **237** | 161 - 336 | **0.7** | 0.5 - 0.9 |
| CACTA | **101** | 82.3 - 122.7 | **655** | 533 - 795 | **1.8** | 1.5 - 2.2 |
| hAT | **3** | 0.6 - 8.8 | **74** | 15 - 215 | **0.2** | 0 - 0.6 |
|  |  |  |  |  |  |  |
| ***O. punctata*[BB; 425 Mbp]** | |  |  |  |  |  |
| *ty-3*-*gypsy* | **642** | 593.3 - 693.6 | **6991** | 6461 - 7554 | **16.5** | 15.2 - 17.8 |
| *ty-1*-*copia* | **109** | 89.5 - 131.5 | **2492** | 2046 - 3006 | **5.9** | 4.8 - 7.1 |
| retrotransposon | **169** | 144.5 - 196.5 | **2493** | 2131 - 2899 | **5.9** | 5 - 6.8 |
| LINE | **26** | 17 - 38.1 | **898** | 587 - 1316 | **2.1** | 1.4 - 3.1 |
| Helitron | **32** | 21.9 - 45.2 | **327** | 224 - 462 | **0.8** | 0.5 - 1.1 |
| CACTA | **160** | 136.2 - 186.8 | **1385** | 1179 - 1617 | **3.3** | 2.8 - 3.8 |
| hAT | **8** | 3.5 - 15.8 | **269** | 116 - 531 | **0.6** | 0.3 - 1.2 |
|  |  |  |  |  |  |  |
| ***O. officinalis*[CC; 651 Mbp]** | |  |  |  |  |  |
| *ty-3*-*gypsy* | **1518** | 1442.6 - 1596.3 | **16188** | –15384-17023 | **24.9** | 23.6-26.1 |
| *ty-1*-*copia* | **192** | 165.8 - 221.2 | **4232** | –3655-4875 | **6.5** | 5.6-7.5 |
| retrotransposon | **320** | 285.9 - 357.1 | **4600** | –4109-5132 | **7.1** | 6.3-7.9 |
| LINE | **23** | 14.6 - 34.5 | **755** | –478-1133 | **1.2** | 0.7-1.7 |
| Helitron | **19** | 11.4 - 29.7 | **191** | –115-298 | **0.3** | 0.2 - 0.5 |
| CACTA | **307** | 273.6 - 343.3 | **2610** | –2327-2919 | **4.0** | 3.6-4.5 |
| hAT | **54** | 40.6 - 70.5 | **1730** | –1299-2257 | **2.7** | 2.0-3.5 |
|  |  |  |  |  |  |  |
| ***O. minuta*[BBCC; 1124 Mbp]** |  |  |  |  |  |  |
| *ty-3*-*gypsy* | **2296** | 2203 - 2391.9 | **21722** | 20843 - 22629 | **19.3** | 18.5 - 20.1 |
| *ty-1*-*copia* | **482** | 439.9 - 527 | **9451** | 8626 - 10334 | **8.4** | 7.7 - 9.2 |
| retrotransposon | **498** | 455.2 - 543.7 | **6356** | 5810 - 6940 | **5.7** | 5.2 - 6.2 |
| LINE | **64** | 49.3 - 81.7 | **1873** | 1443 - 2392 | **1.7** | 1.3 - 2.1 |
| Helitron | **52** | 38.8 - 68.2 | **463** | 345 - 607 | **0.4** | 0.3 - 0.5 |
| CACTA | **515** | 471.5 - 561.5 | **3883** | 3555 - 4233 | **3.4** | 3.2 - 3.8 |
| hAT | **54** | 40.6 - 70.5 | **1542** | 1159 - 2013 | **1.4** | 1 - 1.8 |
|  |  |  |  |  |  |  |
| ***O. alta*[CCDD; 1008 Mbp]** |  |  |  |  |  |  |
| *ty-3*-*gypsy* | **1418** | 1345.1 - 1493.8 | **14992** | 14222 - 15793 | **14.9** | 14.1 - 15.7 |
| *ty-1*-*copia* | **403** | 364.6 - 444.3 | **8846** | 8003 - 9753 | **8.8** | 7.9 - 9.7 |
| retrotransposon | **599** | 552 - 648.9 | **8548** | 7877 - 9261 | **8.5** | 7.8 - 9.2 |
| LINE | **61** | 46.7 - 78.4 | **2002** | 1531 - 2571 | **2.0** | 1.5 - 2.6 |
| Helitron | **33** | 22.7 - 46.3 | **328** | 226 - 461 | **0.3** | 0.2 - 0.5 |
| CACTA | **323** | 288.7 - 360.2 | **2721** | 2432 - 3034 | **2.7** | 2.4 - 3 |
| hAT | **58** | 44 - 75 | **1857** | 1410 - 2401 | **1.8** | 1.4 - 2.4 |
|  |  |  |  |  |  |  |
| ***O. australiensis* [EE: 965 Mbp]** | |  |  |  |  |  |
| *ty-3*-*gypsy* | **2619** | 2519.6 - 2721.3 | **27616** | 26569 - 28695 | **28.6** | 27.5 - 29.7 |
| *ty-1*-*copia* | **724** | 672.2 - 778.7 | **15994** | 14850 - 17203 | **16.6** | 15.4 - 17.8 |
| retrotransposon | **386** | 348.4 - 426.5 | **5510** | 4974 - 6088 | **5.7** | 5.2 - 6.3 |
| LINE | **63** | 48.4 - 80.6 | **2099** | 1613 - 2685 | **2.2** | 1.7 - 2.8 |
| Helitron | **6** | 2.2 - 13.1 | **59** | 22 - 129 | **0.1** | 0 - 0.1 |
| CACTA | **367** | 330.4 - 406.5 | **3078** | 2771 - 3409 | **3.2** | 2.9 - 3.5 |
| hAT | **52** | 38.8 - 68.2 | **1690** | 1262 - 2216 | **1.8** | 1.3 - 2.3 |
|  |  |  |  |  |  |  |
| ***O. brachyantha*[FF; 362 Mbp]** | |  |  |  |  |  |
| *ty-3*-*gypsy* | **266** | 235 - 300 | **2280** | 2015 - 2572 | **6.3** | 5.6 - 7.1 |
| *ty-1*-*copia* | **84** | 67 - 104 | **1500** | 1196 - 1857 | **4.1** | 3.3 - 5.1 |
| retrotransposon | **79** | 62.5 - 98.5 | **915** | 725 - 1141 | **2.5** | 2 - 3.2 |
| LINE | **12** | 6.2 - 21 | **321** | 166 - 561 | **0.9** | 0.5 - 1.6 |
| Helitron | **1** | 0 - 5.6 | **8** | 0 - 45 | **0.0** | 0 - 0.1 |
| CACTA | **28** | 18.6 - 40.5 | **191** | 127 - 276 | **0.5** | 0.4 - 0.8 |
| hAT | **8** | 3.5 - 15.8 | **209** | 90 - 412 | **0.6** | 0.2 - 1.1 |
|  |  |  |  |  |  |  |
| ***O. granulata*[GG; 882 Mbp]** | |  |  |  |  |  |
| *ty-3*-*gypsy* | **3050** | 2942.7 - 3160.2 | **32269** | 31133 - 33435 | **36.6** | 35.3 - 37.9 |
| *ty-1*-*copia* | **317** | 283.1 - 353.9 | **7008** | 6258 - 7824 | **7.9** | 7.1 - 8.9 |
| retrotransposon | **311** | 277.4 - 347.6 | **4451** | 3970 - 4974 | **5.0** | 4.5 - 5.6 |
| LINE | **19** | 11.4 - 29.7 | **632** | 380 - 987 | **0.7** | 0.4 - 1.1 |
| Helitron | **11** | 5.5 - 19.7 | **109** | 55 - 196 | **0.1** | 0.1 - 0.2 |
| CACTA | **28** | 18.6 - 40.5 | **236** | 157 - 341 | **0.3** | 0.2 - 0.4 |
| hAT | **52** | 38.8 - 68.2 | **1687** | 1260 - 2212 | **1.9** | 1.4 - 2.5 |
|  |  |  |  |  |  |  |
| ***O. ridleyi*[HHJJ; 1283 Mbp]** |  |  |  |  |  |  |
| *ty-3*-*gypsy* | **2149** | 2059.1 - 2241.8 | **19832** | 19002 - 20689 | **15.5** | 14.8 - 16.1 |
| *ty-1*-*copia* | **509** | 465.7 - 555.2 | **9757** | 8927 - 10642 | **7.6** | 7 - 8.3 |
| retrotransposon | **971** | 910.9 - 1034 | **12097** | 11348 - 12883 | **9.4** | 8.8 - 10 |
| LINE | **43** | 31.1 - 57.9 | **1233** | 892 - 1661 | **1.0** | 0.7 - 1.3 |
| Helitron | **10** | 4.8 - 18.4 | **87** | 42 - 160 | **0.1** | 0 - 0.1 |
| CACTA | **432** | 392.2 - 474.7 | **3176** | 2883 - 3490 | **2.5** | 2.2 - 2.7 |
| hAT | **54** | 40.6 - 70.5 | **1511** | 1135 - 1971 | **1.2** | 0.9 - 1.5 |
|  |  |  |  |  |  |  |
| ***O. coarctata*[HHKK]*** | |  |  |  |  |  |
| *ty-3*-*gypsy* | **737** | 684.7 - 792.2 | **7930** | 7368 - 8523 | **6.2** | 5.8 - 6.7 |
| *ty-1*-*copia* | **709** | 657.8 - 763.2 | **15866** | 14719 - 17078 | **12.4** | 11.5 - 13.3 |
| retrotransposon | **260** | 229.4 - 293.6 | **3778** | 3333 - 4267 | **2.9** | 2.6 - 3.3 |
| LINE | **55** | 41.4 - 71.6 | **1843** | 1388 - 2399 | **1.4** | 1.1 - 1.9 |
| Helitron | **19** | 11.4 - 29.7 | **192** | 116 - 300 | **0.1** | 0.1 - 0.2 |
| CACTA | **79** | 62.5 - 98.5 | **677** | 536 - 844 | **0.5** | 0.4 - 0.7 |
| hAT | **10** | 4.8 - 18.4 | **327** | 157 - 601 | **0.3** | 0.1 - 0.5 |
|  |  |  |  |  |  |  |

## Additional file 1 - Occurrences per single Mbp for the major classes of repeats in different *Oryza* genomes

A) The absolute number of significant hits to the *Oryza* repeat data bases. C: Estimated number of occurrences calculated using the equation from Hawkins et al (2006) [29]. E: The number normalized to 1 Mbp. Columns B, D and F are the confidence intervals calculated assuming a Poisson distribution of repeats in the genome, calculated for values in columns A, C and E respectively. The mean length used for different repeats were as follows: *Ty-3*-*gypsy* elements: 12Kbp; *Ty-1*-*copia* elements: 5.5 Kbp; others LTR-RTs not classified: 8.75 Kbp; LINEs: 3.5 kbp; Helitrons (complete autonomous): 12.8 Kbp; CACTA: 15.2 Kbp; hAT: 3.6 Kbp

*In this case all the calculations are based on a rough estimate of the genome size of this species: the real value is unknown, we therefore used the value estimated for *O. ridleyi* [HHJJ; 1283 Mb], which is also an allotetraploid species and shares the HH genome type with *O. coarctata*.
